# The Endowment of Research

**Published:** 1898-12-31

**Authors:** 


### The Hospital

A Journal of

The Medical Sciences and Hospital Administration.

Vol. XXV.—No. 640.] Edited by Sir Henry Burdett, K.C.B., and Solomon C. Smith, M.D., M.R.C.P. [Dec. 31, 1898.

### The Endowment of Research.

The munificence of Lord Iveagh has made the first great step in this country towards the endowment of research for scientific purposes, and it is not too much to expect that, even within a very few years, this research may bear fruit in the shape of knowledge calculated to prolong human life and to diminish human suffering. We are informed that his lordship, in the first instance anonymously and through an intermediary, sought advice from a very high medical authority as to the way in which money could most usefully be expended for the promotion of scientific work of the highest character, and of such a nature as to afford promise of results directly beneficial to the human race. He was advised that the Jenner Institute of Preventive Medicine was in possession of every facility for the indicated purposes, except the means of meeting the required expenditure, and hence that he would, by supporting it, be saved the cost of buildings, or difficulty in bringing together a staff of competent workers, with the incidental advantage of avoiding even the appearance of opposition between two institutions aiming at similar objects. Lord Iveagh upon this asked permission to inspect the Institute, and in consequence of this visit, although he had not then fully made up his mind with regard to the employment of his principal donation, he gave £5,000 as an evidence of his complete approval of what he had seen, and, after a short period of further consideration and inquiry, he made a definite offer to Lord Lister and the Council of the Institute of an endowment of £200,000, subject to certain conditions which were calculated to secure the permanent application of the income to the purposes which he desired to serve. One of the conditions was the appointment of trustees with certain powers, and, after discussion, it was arranged that these powers should be limited in the future by an appeal to the President of the Royal Society for the time being in the event of any disagreement or difference of opinion between the trustees and the Council of the Institute. As thus modified the offer was thankfully accepted, and then Lord Iveagh, after calculating probable future requirements in the way of apparatus, salaries, and other expenditure, came to the conclusion that the income of £6,000 a year, which might be expected from his donation, would be hardly sufficient, and volunteered to increase his gift to a quarter of a million, at which amount it is now to stand.

The methods of higher education which have prevailed in Great Britain until within the last few years have been of such a character as to leave those who have been subjected to them almost wholly outside of the progress of physical, and wholly outside that of biological, science. It would not be too much to say that, with a few exceptions, a Master of Arts who is more than forty would not be likely to know more about the physics of electric lighting than the servant who turned on the current in the evening, or more about electric telegraphy than the messenger boy who brought a red envelope to the door. The institution of a Science Tripos, and the unconscious influence of surroundings, have done something, and will in time do more, to modify this condition; but in the department of biology the ignorance of the public is still general and profound, and forms the secure basis upon which every kind of quackery is erected, from homœopathy to "Christian scientism," or from "hypnotism" to "psychical research." The public has never yet been enabled to distinguish between philosophers and quacks; and, with pathetic simplicity, regards the discoveries of the former as a justification for putting trust in the pretensions of the latter. A philosopher has demonstrated the dependence of tubercle upon a bacillus, and the public gape and say that it is all very wonderful, although they will not take the trouble to kill the bacillus in the milk which they give to their children. But, when a quack makes a mess which he calls "tuberculinum," and says that it will cure consumption, the public run after him, and swallow the stuff. In the phrase which was Faraday's especial *bete noir*, they would say it "stands to reason" that "tubercle" should be destroyed by "tuberculinum," inasmuch as, but for this power, "tuberculinum" would be called something else. May we not hope that the gift of a quarter of a million of money for the promotion of biological research, and for the consequent confusion of ignorance and quackery, may give pause to some of the victims of the latter, and may induce many to think that a pursuit which, in the eyes of a man as much distinguished for his shrewdness as for his philanthropy, has been thought worthy of such encouragement, must certainly be also worthy of a high place in the temple of knowledge, and of the best attention of those upon whom it devolves to control the future education of their countrymen.

